# Frailty in Older Adults Is Associated With Plasma Concentrations of Inflammatory Mediators but Not With Lymphocyte Subpopulations

**DOI:** 10.3389/fimmu.2018.01056

**Published:** 2018-05-16

**Authors:** Diego Marcos-Pérez, María Sánchez-Flores, Ana Maseda, Laura Lorenzo-López, José C. Millán-Calenti, Johanna M. Gostner, Dietmar Fuchs, Eduardo Pásaro, Blanca Laffon, Vanessa Valdiglesias

**Affiliations:** ^1^Universidade da Coruña, DICOMOSA Group, Department of Psychology, Area of Psychobiology, Faculty of Education Sciences, A Coruña, Spain; ^2^Department of Cell and Molecular Biology, Faculty of Sciences, Universidade da Coruña, A Coruña, Spain; ^3^Gerontology Research Group, Instituto de Investigación Biomédica de A Coruña (INIBIC), Universidade da Coruña, A Coruña, Spain; ^4^Division of Biological Chemistry, Biocenter, Innsbruck Medical University, Innsbruck, Austria; ^5^ISPUP-EPIUnit, Universidade do Porto, Porto, Portugal

**Keywords:** C-reactive protein, interleukin 6, frailty, inflammaging, lymphocyte subpopulations, tumor necrosis factor α, soluble TNFα receptor II

## Abstract

Frailty denotes a multidimensional syndrome that gives rise to vulnerability to stressors and leads to an increase of the age-related decline of different physiological systems and cognitive abilities. Aging-related alterations of the immune system may compromise its competence culminating in a chronic low-grade inflammation state. Thus, it has been proposed that frailty is associated with alterations in the concentration of pro-inflammatory molecules and in different lymphocyte subpopulations. To provide further support to the validity of that hypothesis, we conducted a cross-sectional study in a population of Spanish older adults (*N* = 259, aged 65 and over) classified according to their frailty status. Biomarkers analyzed included percentages of several lymphocyte subsets and several inflammation mediators, namely concentrations of interleukin 6 (IL6), C-reactive protein (CRP), tumor necrosis factor α (TNFα), and 75 kDa soluble TNFα receptor II (sTNF-RII). Reference ranges for the inflammation mediators were established for the first time in robust older adults. A significant increase in the CD4^+^/CD8^+^ ratio and a significant decrease in the % CD19^+^ cells were observed in the frail group. Progressive increases with frailty severity were obtained in all inflammatory mediator concentrations, especially notable for IL6 and sTNF-RII. Area under the receiver-operating characteristic curve obtained for sTNF-RII (0.90, 95% CI 0.85–0.94, *P* < 0.001) indicates a high accuracy in the predictive value of this biomarker for frailty. Although results from the current study revealed limited strength associations between frailty and the lymphocyte subsets assessed, data obtained for the inflammatory mediators provide further support to involvement of inflammaging in frailty status in older adults.

## Introduction

Nowadays, world population is experiencing an unstoppable aging situation due to the increasing life expectancy and low birth rates, and this trend is evident from the most developed countries to the lowest income regions (1). The 2015 Aging Report by the European Commission has estimated, comparing European population in 2013 and 2060, that young people (aged 0–14) will remain constant (around 15%), that people aged 15–64 will decline slightly from 66 to 57%, but older adults will increase notably: from 18 to 28% those aged 65 and over, and from 5 to 12% those aged 80 and over; this last group will almost become as numerous as the young population in 2060 (2). This rise in the older population, together with the associated sanitary, social, and economic implications, has increased the global interest in the study of aging processes and age-related conditions (3).

Due to the fact that aging manifestations are very heterogeneous, chronological age is not a proper indicator of aging. In this context, the term frailty has been coined as a more precise measurement of aging signs and symptoms. Frailty denotes a multidimensional syndrome of loss of energy, physical ability, cognition, and health that gives rise to vulnerability to stressors (4). This vulnerability leads to a significant increase of the age-related decline of different physiological systems and cognitive abilities, and eventually to comorbidity, disability, hospitalization, and death in older adults (5). Frailty-associated physiological dysregulation involves multi-organ systems including the musculoskeletal, immune, endocrine, hematologic, nervous, and cardiovascular systems (6, 7). Moreover, the existence of an association between frailty and the development of several age-related diseases, such as cardiovascular disease (8), cancer (9), osteoporosis (10), and Alzheimer’s disease (11) is currently accepted.

One of the most commonly used criteria to assess frailty are those developed by Linda Fried and colleagues (12). Due to their simplicity and ease of implementation, it is the most widely used in clinic and research on frailty. Definition according to these criteria represents a phenotypic description of frailty based on the presence or absence of five very specific components related to physical fitness and metabolism (muscle weakness, low gait speed, unintentional weight loss, exhaustion, and low physical activity). Based on Fried’s definition, the prevalence of frailty in community-dwelling Spanish older populations is 3.7–16.3% (13–15), but it can reach 68.8% in institutionalized older people (16). Since it has been demonstrated that frailty is potentially preventable and can be even reverted in its primary stages (17, 18), early detection of frail subjects is critical.

Inflammation is a necessary response of the immune system to different harmful conditions such as infection and injury. This acute and transient immune response resulting in elevated production of cytokines and acute phase proteins facilitates the repair, turnover, and adaptation of many tissues. But when inflammation becomes chronic, often associated with aging or age-related diseases, it seems to have detrimental effects (19). Aging-related alterations of the immune system that compromise its competence are defined as “immunosenescence,” phenomenon partially responsible for increased autoimmunity, raised prevalence, and severity of infectious diseases, and lower efficacy of vaccination in older adults (20). The main feature of immunosenescence is the change in the cellular composition of the T-cell compartment, including a decrease in the number of naive phenotype cells, in association with an increase in the number of memory phenotype cells, all of which culminate a pro-inflammatory state, called “inflammaging,” diminishing the immune system capacity to respond to new antigens (21). This chronic low-grade inflammation is characterized by increases in the levels of pro-inflammatory cytokines such as interleukin 6 (IL6), tumor necrosis factor alpha (TNFα), and its soluble receptors [sTNF-RI and 75 kDa soluble TNFα receptor II (sTNF-RII)], as well as acute phase proteins such as C-reactive protein (CRP), which impair the maintenance of immunological homeostasis (22).

Inflammaging has been postulated to be an underlying mechanism of frailty, acting either directly or indirectly through its negative influence on other physiological systems (23, 24). Thus, studies testing the hypothesis that frailty is associated with alterations in the concentration of immune activation markers, pro-inflammatory molecules, and in different lymphocyte subpopulations are increasing in the last years (25–29), although clear definitive conclusions have not been reached yet.

Hence, in order to provide further support to the validity of that hypothesis and to increase the body of evidence connecting frailty within inflammaging and immunosenescence biomarkers, we conducted a cross-sectional study in a population of older adults (*N* = 259, aged 65 and over) classified according to their frailty status following Fried’s criteria (12). Biomarkers analyzed included percentages of several lymphocyte subsets (CD3^+^, CD4^+^, CD8^+^, CD19^+^, and CD16^+^56^+^), and concentrations of IL6, CRP, sTNF-RII, and TNFα. Besides, the influence of clinical parameters, namely, nutritional and functional status, was also explored.

## Materials and Methods

### Ethics Statement

Ethical approval was obtained from the University of A Coruña Ethics Committee (reference number CE 18/2014). The study was conducted according to the Helsinki Declaration and International Conference of Harmonization guidelines. Written informed consent was obtained from all study participants, or their relatives in case of inability.

### Study Subjects

In total, 259 individuals aged 65–102 years were recruited from Galicia, North-western Spain. They were contacted through associations of older or retired people, day-care centers, and nursing homes. Table 1 shows the general characteristics of the study population, classified according to the frailty status. The small number of current smokers and ex-smokers (*N* = 5 and *N* = 49, respectively) motivated to join them all in a new category, “ever smokers.” Similarly, malnourished individuals (*N* = 14) and individuals at risk of malnutrition (*N* = 80) were included together in a single category.

**Table 1:** Description of the study population.

	Non-frail	Pre-frail	Frail	*P*
Total individuals *N* (%)	40 (15.4)	131 (50.6)	88 (34.0)	
**Gender *N* (%)**				
Males	27 (67.5)	36 (27.5)	22 (25.0)	<0.001^b^
Females	13 (32.5)	95 (72.5)	66 (75.0)	
Age (years-old)^a^	73.2 ± 5.5 (65–85)	77.05 ± 7.7 (65–100)	85.8 ± 7.9 (65–102)	<0.001^c^
65–69	13 (32.5)	29 (22.1)	2 (2.3)	<0.001^b^
70–74	11 (27.5)	26 (19.9)	4 (4.6)	
75–79	10 (25.0)	24 (18.3)	13 (14.9)	
80–84	5 (12.5)	27 (20.6)	14 (16.1)	
≥85	1 (2.5)	25 (19.1)	54 (62.1)	
**Smoking habit *N* (%)**				
Non-smokers	22 (55.0)	102 (78.5)	76 (90.5)	<0.001^b^
Ever smokers	18 (45.0)	28 (21.5)	8 (9.5)	
No. cigarettes/day^a^	16.1 ± 8.8 (3–40)	15.7 ± 13.9 (2–60)	31.4 ± 15.7 (2–60)	0.020^c^
Years smoking^a^	19.4 ± 9.1 (10–34)	30.4 ± 18.7 (4–66)	29.3 ± 18.2 (6–52)	0.154^c^
**Comorbidity *N* (%)**				
No comorbidity	34 (85.0)	92 (70.2)	52 (59.8)	0.015^b^
Comorbidity	6 (15.0)	39 (29.8)	35 (40.2)	
**Living conditions *N* (%)**				
Family home	40 (100.0)	113 (86.3)	5 (5.7)	<0.001^b^
Family home + daycare center	–	4 (3.1)	23 (26.1)	
Nursing home	–	14 (10.6)	60 (68.2)	

*^a^Mean ± SD (range)*.

*^b^Chi-square test (bilateral)*.

*^c^ANOVA test (bilateral)*.

Participants were individually assessed at the centers, by interviewers specially trained in clinical evaluation to unify criteria, and all completed a questionnaire to assess demographic, lifestyle, and medical information. Participants were excluded if they did not possess the necessary skills to be assessed or denied signing the informed consent. Exclusion criteria also included taking medications included in the Anatomical Therapeutic Chemical category L (antineoplastic or immunomodulating agents) (30), and having any chronic infection, autoimmune disease, or cancer, since these conditions are directly related to immune system dysfunctions and, consequently, they could act as confounders.

Whole blood samples were obtained by venipuncture and collected into vacutainer tubes containing ethylenediaminetetraacetic acid between 9:30 h and 12:30 h and transported immediately to the lab. Fresh whole blood samples were used for the analysis of lymphocyte subsets. After centrifugation at 2,300 rpm for 10 min to obtain plasma, samples were aliquoted and stored at −80°C until analysis of inflammatory mediators. All samples were coded at the moment of collection to ensure a “blind” study.

### Frailty Status

Frailty status of each participant was assessed according to the five phenotypic criteria proposed by Fried et al. (12). These criteria are based on the presence or absence of specific phenotypic components:

(i) Unintentional weight loss (i.e., not due to dieting or exercise): at least 4.5 kg in the past year,
(ii) Self-reported exhaustion: identified by two questions from the Spanish version (31) of the modified 10-item Center for Epidemiological Studies-Depression (CES-D) scale (32),
(iii) Weakness: grip strength in the lowest 20% at baseline, adjusted for gender and body mass index,
(iv) Slow walking speed: the slowest 20% at baseline, based on time to walk 15 ft, adjusting for gender and standing height, and
(v) Low physical activity: the lowest 20% at baseline, based on a weighted score of kilocalories expended per week, calculated according to the Spanish validation (33) of the Minnesota Leisure Time Activity questionnaire (34) according to each participant’s report, and adjusting for gender.

Frailty was defined as the presence of three or more of these characteristics, pre-frailty in case of one or two of them present, and the absence of all five determined a non-frail state.

### Comorbidity

General comorbidity and number of comorbid diseases were assessed by means of the Charlson’s comorbidity index (35). For each subject, an age-adjusted score was computed, coding the absence of comorbid diseases as 0, and the presence as 1–6.

### Lymphocyte Subpopulations

Three-color direct immunofluorescence surface marker analysis was carried out by flow cytometry as previously described (36) to determine peripheral blood lymphocyte phenotypes as follows: T lymphocytes (CD3^+^), T-helper lymphocytes (CD3^+^ and CD4^+^), T-cytotoxic lymphocytes (CD3^+^ and CD8^+^), B lymphocytes (CD19^+^), and NK cells (CD3^−^ and CD16^+^56^+^). Samples were analyzed in a FACScalibur flow cytometer (Becton Dickinson) using CellQuest Pro software (Becton Dickinson). Lymphocytes were gated on the basis of size and complexity, and fluorescence data were obtained to determine percentages of the different lymphocyte subsets, acquiring a minimum of 10^4^ events in the lymphocyte window.

### Circulating Inflammatory Molecules

Plasma levels of IL6, CRP, TNFα, and sTNF-RII were measured by quantitative sandwich enzyme-linked immunosorbent assays with commercial kits (all from R&D Systems Inc., Minneapolis, MN, USA), according to the manufacturer’s instructions. Samples required a 100-fold dilution for analysis of CRP, and a 10-fold dilution for analysis of sTNF-RII. Regarding precision of the assays, maximum intra- and inter-assay coefficients of variation were 4.2 and 6.4% for IL6, 8.6 and 7.0% for CRP, 3.0 and 8.4% for TNFα, and 4.8 and 5.1% for sTNF-RII, respectively.

Tumor necrosis factor α concentration could only be evaluated in specimens from 88 individuals (20 non-frail, 38 pre-frail, and 30 frail), of whom sufficient residual plasma volume was available. Spectrophotometric measurements were conducted in a Powerwave X microplate reader (Bio-Tek Instruments) equipped with kinetic analysis software (KC4 v.2.5, Bio-Tek Instruments).

### Statistical Analysis

A general description of the study population, classified according to the frailty status, was carried out by univariate analysis. Sociodemographic, lifestyle, and clinical characteristics were compared in the three groups of subjects, applying analysis of variance (ANOVA) for continuous variables and Chi-square test for categorical variables (Table 1).

The effect of frailty status on the immune biomarkers determined was preliminarily assessed by ANOVA and Tuckey’s *post hoc* test. Data from % CD3^+^, % CD4^+^, % CD16^+^56^+^, and TNFα followed a normal distribution (Kolmogorov–Smirnov goodness-of-fit test). A log-transformation of the data was applied to % CD8^+^, % CD19^+^, IL6, and CRP to achieve a better approximation to the normal distribution. No improvement was achieved with transformation for CD4^+^/CD8^+^ratio and sTNF-RII, so they were analyzed using the Kruskal–Wallis test with Bonferroni’s correction.

Values of IL6, CRP, sTNF-RII and TNFα obtained in non-frail and pre-frail individuals were used for calculating reference ranges. They were defined as mean ± 2 SD for those parameters following a Gaussian distribution. For data not normally distributed (sTNF-RII), reference range was calculated as the central 95% of the area under the distribution curve (from 2.5 to 97.5%).

Linear regression analysis was applied to estimate the effect of frailty status on the immune parameters. Models were run with log-transformed data, adjusting for age, gender, smoking habit (never/ever smokers) and comorbidity. All results are shown as mean ratios and 95% confidence intervals (95% CI).

Correlation coefficients (Spearman’s rho) were calculated in order to estimate the possible associations between immune biomarkers. Receiver-operating characteristic (ROC) curves were computed to assess the inflammatory biomarkers discriminating ability. Statistical analyses were conducted by means of the STATA/SE software package V. 12.0 (StataCorp LP) and the IBM SPSS software package V. 20 (SPSS, Inc.). Statistical significance was established at a *P* value lower than 0.05.

## Results

Study population comprised 259 older adults, aged from 65 to 102 years; frail subjects were significantly older, with 62% aged over 85 years (Table 1). Female gender was less prevalent in the non-frail group (approximately 1:2), but gender proportions were opposite in the pre-frail and frail groups. Proportion of smokers decreased with increasing frailty severity; frail smokers consumed a higher amount of cigarettes per day, but the number of years smoking was not significantly different among the three groups of smokers. Only 10% of non-frail individuals were at risk of malnutrition, with no one malnourished. The rate of malnourishment increased, and consequently the MNA-SF score decreased, with frailty severity. There were only two subjects (5%) in the non-frail group with ADL dependence, while ADL dependence was present in 94% of frail subjects. Similarly, comorbidity increased with frailty, from 15% in the non-frail group to 40% in the frail group. Regarding living conditions, all non-frail individuals lived at family homes, while nearly 70% of frail participants lived in nursing homes.

**Figure 1:**
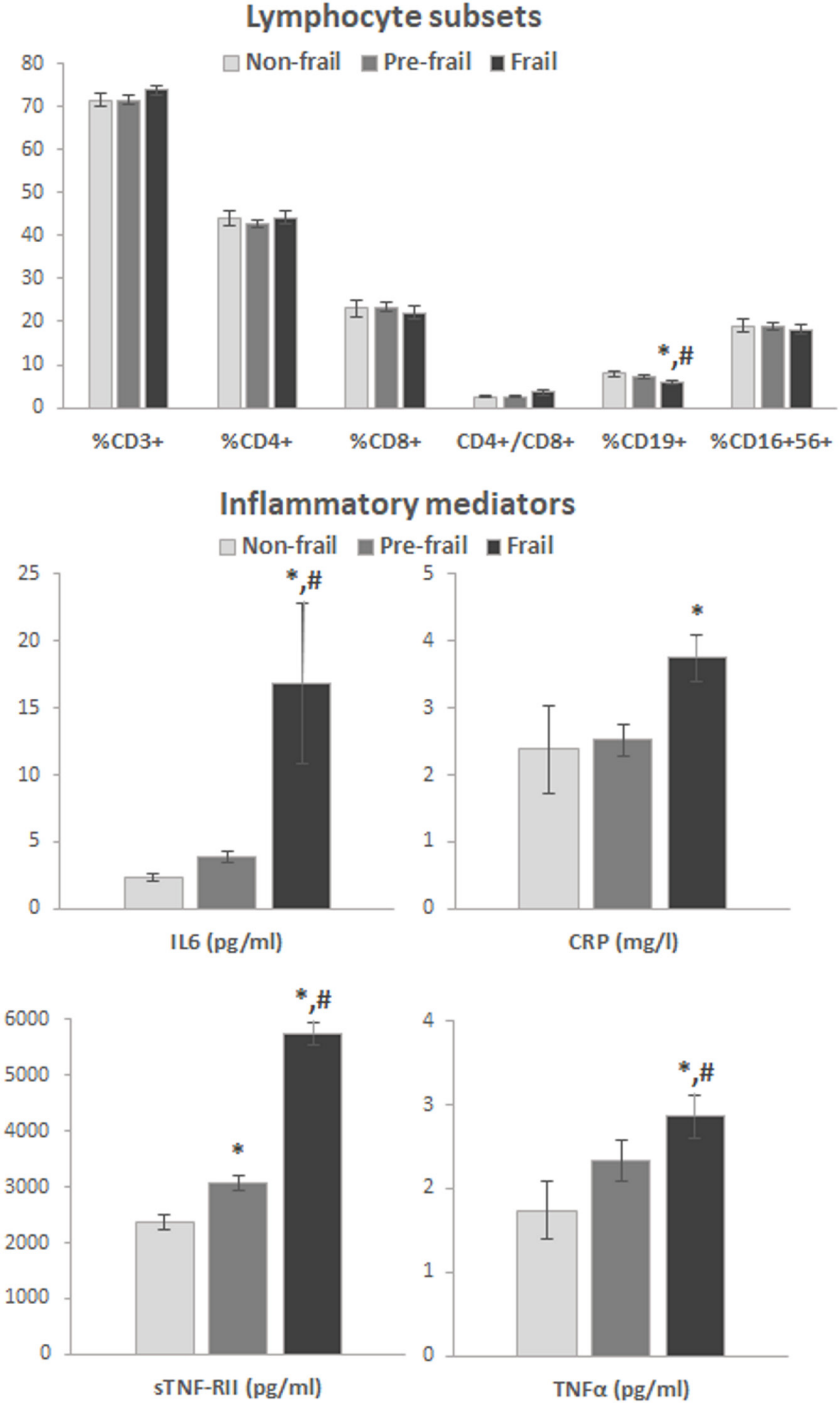
Results of immunological biomarkers in the study group, classified according to frailty status (univariant analysis). Bars represent mean standard error. *Statistically different from non-frail, ^#^statistically different from pre-frail (Tukey’s test or Bonferroni’s correction). Abbreviations: CRP, C-reactive protein, IL6: interleukin 6, TNFα: tumor necrosis factor alpha, sTNF-RII, soluble tumor necrosis factor alpha receptor II.

Figure 1 shows values of the immune parameters determined in the three groups of older adults, with univariate analysis comparison. The only lymphocyte subset showing significant changes was % CD19^+^ cells, which decreased significantly in the group of frail subjects. On the contrary, significant increases with frailty were observed for IL6, CRP, sTNF-RII and TNFα concentrations. No differences were obtained in these markers between non-frail and pre-frail individuals, except for sTNF-RII.

Reference ranges for lymphocyte subpopulations in non-frail older adults were described previously (36). When comparing the current results with those reference ranges, 91–97% of values obtained for the different subsets in the non-frail group, 94–97% of values in the pre-frail group, and 83–97% of values in the frail group fell within the established ranges, with no significant differences between groups for any particular subset. Reference ranges in robust older adults for IL6, CRP, sTNF-RII and TNFα had not been described so far. Thus, concentrations obtained in the non-frail and pre-frail subjects were used for estimating the upper and lower limits of the corresponding reference ranges (Table 2), since no significant differences were observed between these two groups, except in the case of sTNF-RII, where differences with frail individuals were much more remarkable. Percentages of frail subjects with values exceeding the corresponding reference ranges for the different parameters oscillated from 9 to 19%, with no value below the range in any case.

**Table 2:** Reference ranges of the immune biomarkers analyzed, calculated on the basis of results obtained in non-frail and pre-frail subjects.

	*N*	Reference range		% frail subjects out of the reference range	
	
		Lower limit	Upper limit	Below	Above
IL6 (pg/ml)	160	0.20	14.65	0	13.4
CRP (mg/l)	160	0.24	9.90	0	9.3
sTNF-RII (pg/ml)	160	1,322.8	6,563.2	0	18.8
TNFα (pg/ml)	58	0	5.15	0	10.0

*CRP, C-reactive protein; IL6, interleukin 6; TNFα, tumor necrosis factor alpha; sTNF-RII, soluble tumor necrosis factor alpha receptor II*.

In the analysis of the associations between immune biomarkers, notable and significant correlations were obtained between CRP and IL6 (*r* = 0.405, *P* < 0.001), between CRP and sTNF-RII (*r* = 0.339, *P* < 0.001), between sTNF-RII and TNFα (*r* = 0.433, *P* < 0.001), between TNFα and IL6 (*r* = 0.344, *P* < 0.01), and between IL6 and sTNF-RII (*r* = 0.250, *P* < 0.001).

Results obtained from the multivariate statistical analyses regarding frailty status are shown in Table 3. A significant increase in the CD4^+^/CD8^+^ ratio and a significant decrease in the % CD19^+^ cells were observed in the frail group. Moreover, and according to the results from the univariate analysis, progressive increases with frailty severity were obtained in all inflammatory mediator concentrations; being especially remarkable the 70% increase of IL6 and the twofold increase of sTNF-RII in the frail subjects with regard to the non-frail participants.

**Table 3:** Effect of frailty status on lymphocyte subsets and inflammatory mediators; models adjusted by age, sex, smoking habit, and comorbidity.

	Mean ratio	95% CI	Mean ratio	95% CI	Mean ratio	95% CI	Mean ratio	95% CI	Mean ratio	95% CI
	
	% CD3^+^		% CD4^+^		% CD8^+^		CD4^+^/CD8^+^		% CD19^+^	
**Frailty status**										
Non-frail	1.00		1.00		1.00		1.00		1.00	
Pre-frail	1.00	(1.00–1.17)	1.09	(0.86–1.38)	1.00	(0.81–1.24)	1.09	(0.77–1.55)	0.82	(0.65–1.04)
Frail	0.98	(0.94–1.03)	1.31	(0.98–1.76)	0.82	(0.64–1.06)	1.66*	**(1.09–2.53)**	0.73*	**(0.55–0.97)**

	**% CD16^+^56^+^**		**IL6**		**CRP**		**sTNF-RII**		**TNFα**	

Non-frail	1.00		1.00		1.00		1.00		1.00	
Pre-frail	1.07	(0.85–1.35)	1.15	(0.75–1.75)	1.19	(0.80–1.75)	1.19*	**(1.03–1.38)**	1.60^†^	(0.96–2.66)
Frail	0.92	(0.70–1.21)	1.70*	**(1.03–2.83)**	1.54^†^	(0.96–2.46)	2.00**	**(1.68–2.39)**	1.68^‡^	(0.92–3.09)

*CI, confidence interval; CRP, C-reactive protein; IL6, interleukin 6; TNFα, tumor necrosis factor alpha; sTNF-RII, soluble tumor necrosis factor alpha receptor II*.

*P < 0.05; **P < 0.01; ^†^P = 0.071; ^‡^P = 0.090.

*Bold font means statistically significant data*.

**Figure 2:**
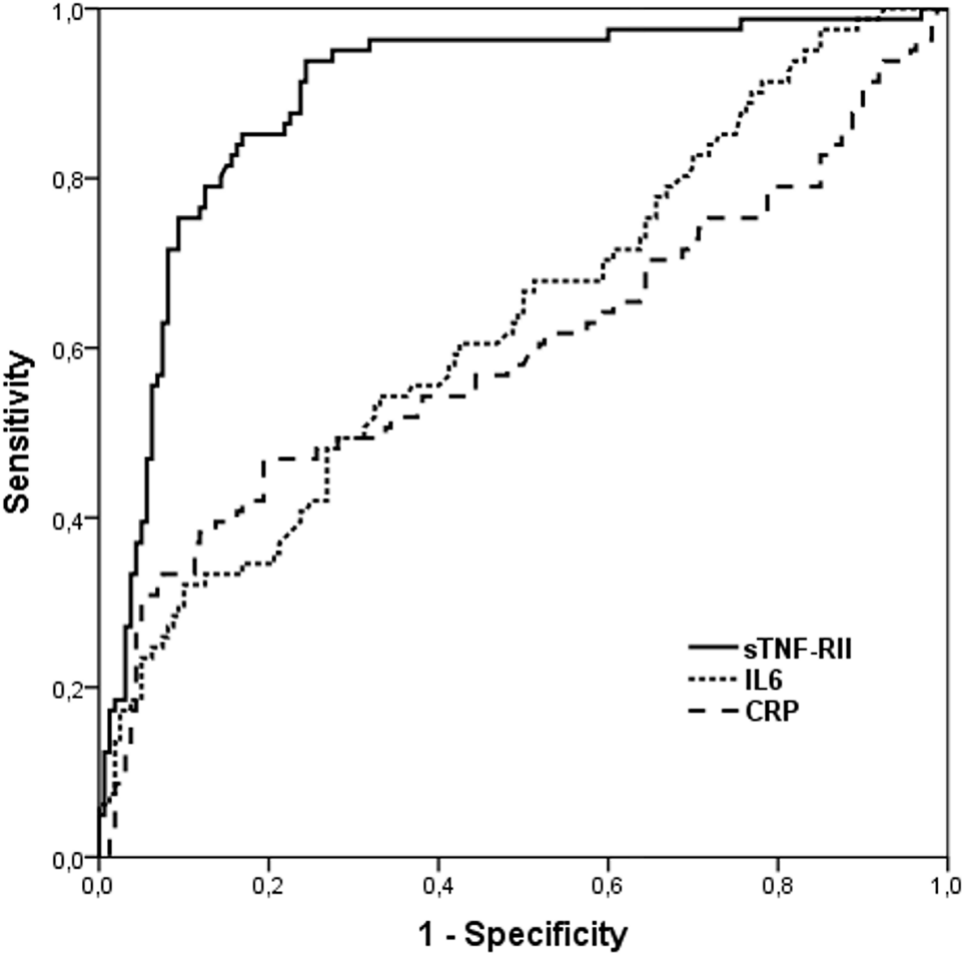
Receiver-operating characteristic (ROC) curves for IL6, CRP, and sTNF-RII to predict frailty. Abbreviations: CRP, C-reactive protein; IL6, interleukin 6; sTNF-RII, soluble tumor necrosis factor alpha receptor II.

Figure 2 shows the ROC curves computed to test the predictive value of the inflammatory molecules for frailty (except for TNFα due to the much lower data available), using the non-frail group as the standard. Areas under the curves obtained were 0.64 (95% CI 0.56–0.71, *P* < 0.01) for IL6, 0.60 (95% CI 0.52–0.68, *P* < 0.05) for CRP, 0.90 (95% CI 0.85–0.94, *P* < 0.001) for sTNF-RII, and 0.66 (95% CI 0.54–0.77, *P* < 0.05) for TNFα. A sTNF-RII concentration of 3,461.3 pg/ml had the optimal predictive value for frailty, with a sensitivity of 0.94 and a specificity of 0.76.

## Discussion

Use of biomarkers as feasible endpoints has been proposed for frailty identification (37), since they would provide a more accurate detection of frail subjects in early stages, when frailty can still be potentially reverted. For the development of frailty-related biomarkers, physiological processes disturbed in frailty status must be explored. Due to the proposed relationship between immune system alterations and frailty (38), pro-inflammatory molecule concentrations and different lymphocyte subpopulation rates could be suitable biomarkers that provide useful information for early identification of frailty. Thus, in this study, a set of immune biomarkers was assessed in a population of Spanish older adults (40 non-frail, 131 pre-frail, and 88 frail), and the possible association between biomarkers and frailty status was evaluated.

Since specific reference ranges for lymphocyte subset rates were previously reported for older subjects with frailty discarded (36), results from the current study were compared with those ranges. Most values obtained in the three population groups fell within the corresponding reference ranges, and no differences were observed regarding frailty severity, indicating a poor connection with frailty for all lymphocyte subpopulations. In spite of the previous vast literature analyzing inflammatory mediators in populations of older adults, specific reference ranges for this age group had not been reported so far. Thus, reference ranges for IL6, CRP, sTNF-RII, and TNFα in robust adults (excluding the presence of frailty) aged 65 years and over were established in this study. Percentages of frail subjects presenting concentrations of these biomarkers out of the corresponding reference range oscillated between 9% in the case of CRP and 19% in the case of sTNF-RII. These values were always located above the reference range, showing a clear trend to increase with frailty status.

In which regards association of frailty with immune biomarkers, our results of lymphocyte subsets showed a slight decrease of % CD19^+^ cells in the frail group—both in the univariate analysis and in the linear regression analysis adjusting for age, gender, smoking habit, and comorbidity—and an increase of the CD4^+^/CD8^+^ ratio (*P* < 0.05) in frail subjects in the multivariate analysis, not significant in the univariate analysis. These quite weak results point to a limited strength association of these biomarkers with frailty. Up to now, very few studies have assessed the link between lymphocyte subpopulations and frailty status in older adults. De Fanis et al. (39) found a significant association between increased CD8^+^ and decreased CD4^+^ cell percentages in frail subjects regarding to the non-frail group, although sample size evaluated was very modest (13 frail vs. 13 non-frail participants). Besides, Semba et al. (40) obtained similar results in addition to a subsequent significant decrease in the CD4^+^/CD8^+^ ratio, with a quite larger population size (*N* = 24, 75, and 28 for non-frail, pre-frail, and frail individuals, respectively). None of these studies adjusted for possible confounders in the statistical analysis, what may account in part for the differences with the current study, together with the more restricted sample sizes.

During inflammation, TNFα, IL1, and IL6 are secreted, in that order. IL6 then inhibits the secretion of TNFα and IL1, and activates the production of acute phase reactants from liver (CRP) [reviewed in Ref. (41)]. TNFα membrane receptors are shed by proteolytic cleavage into circulation as soluble TNFα receptors (sTNF-RI and sTNF-RII), which have been shown to be reliable measurements for the *in vivo* activities of TNFα (42). Results from the present study support the idea of an interrelated activation of the entire inflammatory cascade, since TNFα, sTNF-RII, IL6, and CRP concentrations were significantly correlated with one another.

Data obtained in this work showed positive influence of frailty on IL6, CRP, TNFα, and sTNF-RII concentrations. The only study analyzing sTNF-RII concentrations in relation to frailty so far found progressive increase of this biomarker with frailty status; significance was reached in the group of pre-frail subjects (43). Still, considerable amount of literature has accumulated concerning the association of high levels of IL6, TNFα, and CRP with frailty in older adults in cross-sectional studies. Among works assessing all these three biomarkers, some of them reported increases in their concentrations with frailty (26–28, 44, 45) and with increased risk of death (46). On the contrary, other studies did not find such significant associations with frailty (47), or obtained mixed results (significance for some markers and no effect for others) (38, 48). A recent meta-analysis (49) conducted with most abovementioned studies and some others both cross-sectional and longitudinal concluded that, on the basis of cross-sectional studies, frailty and pre-frailty are associated with higher inflammatory parameter levels, in particular, CRP and IL6, but these findings were not confirmed in longitudinal trials, supporting the need of further studies to better understand the role of inflammatory markers in frailty status. Our study confirmed the involvement of chronic inflammation in frailty in later life; particularly strong associations were obtained in the regression analysis for IL6 (70% increase in frail subjects with regard to non-frail participants), and for sTNF-RII (19% increase in pre-frail and twofold increase in frail individuals; all three categories were significantly different). Moreover, area under the ROC curve obtained for sTNF-RII (0.90) indicates a high accuracy in the predictive value of this biomarker for frailty. At concentrations >3,461.3 pg/ml, frail subjects can be identified with quite high sensitivity (0.94) and specificity (0.76).

Numerous studies of older adults showed that levels of several inflammatory mediators increase with age even in apparently healthy individuals and in the absence of acute infection [reviewed in Ref. (50)]. Present results show that frailty status in older adults involves an additional increase in these mediators, beyond that related to aging. Chronic inflammation has been proposed as a key underlying mechanism involved in frailty (23, 24). Inflammatory molecules may directly contribute to frailty or its central components (such as decreased muscle mass, strength, and power, and slowed motor performance). But, as frailty is a multidimensional syndrome, the contribution can also be indirect through other intermediate pathophysiologic processes, i.e., its detrimental effects on other organ systems, such as musculoskeletal and endocrine systems, cardiovascular diseases, and nutritional dysregulation [reviewed in Ref. (51)].

Increasing evidence suggests that frailty is a useful risk assessment tool for pre-surgery evaluation, for overall immune functional decline, in older patients with cardiovascular conditions, or for risk stratification of older patients with cancer [reviewed in Ref. (51)]. Hence, the importance of identifying frailty is undeniable. The current study suggests that sTNF-RII may have clinical applicability as a screening tool for identifying frail subjects, although standardization and replication of these results in other populations is necessary before it can be used to that aim. A primary limitation of this work includes the medical situation of the participants, intrinsically associated with studying older adults. Pathologic conditions were present in most of them, 15% of robust individuals and 40% of frail subjects presented comorbidity, and medications were taken to treat these conditions. Although linear regression analyses were adjusted for comorbidity and exclusion criteria included (i) taking antineoplastic or immunomodulating medications, and (ii) having infections, autoimmune disease, or cancer, the fact that some of the chronic diseases common in older adults, or the associated medications, may have influenced the immune parameters evaluated in this study cannot be ruled out.

## Conclusion

In this work, reference ranges for several inflammation mediators are established for the first time in older adults in the absence of frailty according to Fried’s criteria. Associations found between inflammatory molecules confirm their interrelationship in the immune activation cascade. Although results from the current study revealed limited strength associations between frailty and the lymphocyte subsets assessed, data obtained for the different inflammatory mediators provide additional reinforcement to the widely established hypothesis that inflammaging is involved in frailty status in older adults. Hence, frail subjects present an additional degree of chronic inflammation manifestations than what could be expected only according to the normal aging process. This association was more intensively manifested in IL6 and sTNF-RII. This last biomarker showed a high accuracy for predicting frailty.

## Data Availability

The raw data supporting the conclusions of this manuscript will be made available by the authors, without undue reservation, to any qualified researcher.

## Ethics Statement

Ethical approval was obtained from the University of A Coruña Ethics Committee (reference number CE 18/2014). The study was conducted according to the Helsinki Declaration and International Conference of Harmonization guidelines. Written informed consent was obtained from all study participants, or their relatives in case of inability.

## Author Contributions

Study concept and design: BL, EP, JM-C, and VV. Acquisition of data: AM, LL-L, DM-P, MS-F, JG, and DF. Analysis and interpretation of data: BL, VV, DF, EP, and JM-C. Drafting of the manuscript: BL and VV. Critical revision of the manuscript for important intellectual content: EP, JM-C, AM, LL-L, DM-P, MS-F, JG, and DF.

## Conflict of Interest Statement

The authors declare that the research was conducted in the absence of any commercial or financial relationships that could be construed as a potential conflict of interest.

## References

[1] Cesari M Prince M Thiyagarajan JA De Carvalho IA Bernabei R Chan P Frailty: an emerging public health priority. J Am Med Dir Assoc (2016) 17:188–92. 10.1016/j.jamda.2015.12.016 26805753

[2] European Commission. The 2015 Ageing Report. Underlying Assumptions and Projection Methodologies. Joint Report Prepared by the European Commission (DG ECFIN) and the Economic Policy Committee (AWG). European Economy Series No. 8. Brussels: European Union (2014). 424 p.

[3] Pizza V Agresta A D’Acunto CW Festa M Capasso A. Neuroinflammation and ageing: current theories and an overview of the data. Rev Recent Clin Trials (2011) 6(3):189–203. 10.2174/157488711796575577 21241238

[4] Rockwood K Song X Macknight C Bergman H Hogan DB McDowell I A global clinical measure of fitness and frailty in elderly people. CMAJ (2005) 173:9–13. 10.1503/cmaj.050051 16129869 PMC1188185

[5] Mulero J Zafrilla P Martínez-Cacha A. Oxidative stress, frailty and cognitive decline. J Nutr Health Aging (2011) 15:756–60. 10.1007/s12603-011-0130-5 22089224 PMC12878021

[6] Fried LP Xue QL Cappola AR Ferrucci L Chaves P Varadhan R Nonlinear multisystem physiological dysregulation associated with frailty in older women: implications for etiology and treatment. J Gerontol A Biol Sci Med Sci (2009) 64:1049–57. 10.1093/gerona/glp076 19567825 PMC2737590

[7] Leng SX Xue QL Tian J Huang Y Yeh SH Fried LP Associations of neutrophil and monocyte counts with frailty in community-dwelling disabled older women: results from the Women’s Health and Aging Studies I. Exp Gerontol (2009) 44:511–6. 10.1016/j.exger.2009.05.005 19457449

[8] Libby P Okamoto Y Rocha VZ Folco E. Inflammation in atherosclerosis: transition from theory to practice. Circ J (2010) 74:213–20. 10.1253/circj.CJ-09-0706 20065609

[9] Grivennikov SI Karin M. Inflammatory cytokines in cancer: tumour necrosis factor and interleukin 6 take the stage. Ann Rheum Dis (2011) 70:i104–8. 10.1136/ard.2010.140145 21339211

[10] Lencel P Magne D. Inflammaging: the driving force in osteoporosis? Med Hypotheses (2011) 76:317–21. 10.1016/j.mehy.2010.09.023 20961694

[11] Morales I Farías G MacCioni RB. Neuroimmunomodulation in the pathogenesis of Alzheimer’s disease. Neuroimmunomodulation (2010) 17:202–4. 10.1159/000258724 20134203

[12] Fried LP Tangen CM Walston J Newman AB Hirsch C Gottdiener J Frailty in older adults: evidence for a phenotype. J Gerontol A Biol Sci Med Sci (2001) 56:M146–56. 10.1093/gerona/56.3.M146 11253156

[13] Abizanda P Sánchez-Jurado PM Romero L Martínez-Sánchez E Atienzar-Núñez P Prevalence of frailty in a Spanish elderly population: the frailty and dependence in Albacete study. J Am Geriatr Soc (2011) 59:1356–9. 10.1111/jgs.12916 21751977

[14] García-García FJ Gutiérrez Ávila G Alfaro-Acha A Amor Andres MS De Los Angeles De La Torre Lonza M Escribano Aparicio MV The prevalence of frailty syndrome in an older population from Spain. Toledo Study Heal Aging (2011) 15:852–6. 10.1007/s12603-011-0075-8 PMC12878005 22159772

[15] Maseda A Gómez-Caamaño S Lorenzo-López L López-López R Diego-Diez C Sanluís-Martínez V Health determinants of nutritional status in community-dwelling older population: the VERISAUDE study. Public Health Nutr (2016) 19:2220–8. 10.1017/S1368980016000434 26975221 PMC10270800

[16] González-Vaca J De La Rica-Escuín M Silva-Iglesias M Arjonilla-García MD Varela-Pérez R Oliver-Carbonell JL Frailty in institutionalized older adults from Albacete. The FINAL Study: rationale, design, methodology, prevalence and attributes. Maturitas (2014) 77:78–84. 10.1016/j.maturitas.2013.10.005 24189222

[17] Espinoza SE Junq I Hazuda H. Frailty transitions in the San Antonio longitudinal study of aging. J Am Geriatr Soc (2012) 60:652–60. 10.1111/j.1532-5415.2011.03882 22316162 PMC3325321

[18] Roland KP Theou O Jakobi JM Swan L Jones GR. How do community physical and occupational therapists classify frailty? A pilot study. J Frailty Aging (2014) 3:247–50. 10.14283/jfa.2014.32 27048865

[19] Penninx BWJH Kritchevsky SB Newman AB Nicklas BJ Simonsick EM Rubin S Inflammatory markers and incident mobility limitation in the elderly. J Am Geriatr Soc (2004) 52:1105–13. 10.1111/j.1532-5415.2004.52308.x 15209648

[20] Mahbub S Brubaker AL Kovacs EJ. Aging of the innate immune system: an update. Curr Immunol Rev (2011) 7:104–15. 10.2174/157339511794474181 21461315 PMC3066013

[21] de Araújo AL Silva LCR Fernandes JR Benard G. Preventing or reversing immunosenescence: can exercise be an immunotherapy? Immunotherapy (2013) 5:879–93. 10.2217/imt.13.77 23902557

[22] Petersen AMW Pedersen BK The anti-inflammatory effect of exercise. J Appl Physiol (2005) 98:1154–62. 10.1152/japplphysiol.00164.2004 15772055

[23] Fulop T Larbi A Witkowski JM McElhaney J Loeb M Mitnitski A Aging, frailty and age-related diseases. Biogerontology (2010) 11:547–63. 10.1007/s10522-010-9287-2 20559726

[24] Li H Manwani B Leng SX Frailty, inflammation, and immunity. Aging Dis (2011) 2:466–73. 22396895 PMC3295062

[25] Baylis D Bartlett DB Syddall HE Ntani G Gale CR Cooper C Immune-endocrine biomarkers as predictors of frailty and mortality: a 10-year longitudinal study in community-dwelling older people. Age (Dordr) (2013) 35:963–71. 10.1007/s11357-012-9396-8 22388931 PMC3636387

[26] Collerton J Martin-Ruiz C Davies K Hilkens CM Isaacs J Kolenda C Frailty and the role of inflammation, immunosenescence and cellular ageing n the very old: cross-sectional findings from the Newcastle 85+ study. Mech Ageing Dev (2012) 133:456–66. 10.1016/j.mad.2012.05.005 22663935

[27] Hubbard RE O’Mahony MS Calver BL Woodhouse KW. Plasma esterases and inflammation in ageing and frailty. Eur J Clin Pharmacol (2008) 64:895–900. 10.1007/s00228-008-0499-1 18506436

[28] Hubbard RE O’Mahony MS Savva GM Calver BL Woodhouse KW. Inflammation and frailty measures in older people. J Cell Mol Med (2009) 13:3103–9. 10.1111/j.1582-4934.2009.00733.x 19438806 PMC4516469

[29] Marcos-Pérez D Sánchez-Flores M Maseda A Lorenzo-López L Millán-Calenti JC Strasser B Frailty status in older adults is related to alterations in indoleamine 2, 3-dioxygenase 1 and guanosine triphosphate cyclohydrolase I enzymatic pathways. J Am Med Dir Assoc (2017) 18:1049–57. 10.1016/j.jamda.2017.06.021 28801236

[30] WHO Collaborating Centre for Drug Statistics Methodology. Guidelines for ATC Classification and DDD Assignment 2013. Oslo: WHO Collaborating Centre for Drug Statistics Methodology (2012).

[31] Ruiz-Grosso P Loret de Mola C Vega-Dienstmaier JM Arevalo JM Chavez K Vilela A Validation of the Spanish center for epidemiological studies depression and Zung Self-Rating Depression Scales: a comparative validation study. PloS One (2012) 7:e45413. 10.1371/journal.pone.0045413 23056202 PMC3466285

[32] Radloff LS The CES-D scale: a self-report depression scale for research in the general population. App Psychol Meas (1977) 1:385–401. 10.1177/014662167700100306

[33] Ruiz-Comellas A Pera G Baena-Díez JM Mundet Tudurí X Alzamora Sas T Elosua R Validación de una versión reducida en español del cuestionario de actividad física en el tiempo libre de Minnesota. Rev Esp Salud Publica (2012) 86:495–508. 10.4321/S1135-57272012000500004 23223762

[34] Taylor HL Jacobs DR Jr Schucker B Knudsen J Leon SL Debacker G A questionnaire for the assessment of leisure time physical activities. J Chronic Dis (1978) 3:741–55. 10.1016/0021-9681(78)90058-9 748370

[35] Charlson ME Pompei P Ales KL MacKenzie CR. A new method of classifying prognostic comorbidity in longitudinal studies: development and validation. J Chronic Dis (1987) 40:373–83. 10.1016/0021-9681(87)90171-8 3558716

[36] Valdiglesias V Sánchez-Flores M Maseda A Marcos-Pérez D Millán-Calenti JC Pásaro E Lymphocyte subsets in a population of nonfrail elderly individuals. J Toxicol Environ Health A (2015) 78:790–804. 10.1080/15287394.2015.1051170 26167746

[37] Mitnitski A Collerton J Martin-Ruiz C Jagger C von Zglinicki T Rockwood K Age-related frailty and its association with biological markers of ageing. BMC Med (2015) 13:161. 10.1186/s12916-015-0400-x 26166298 PMC4499935

[38] Lai HY Chang HT Lee YL Hwang SJ. Association between inflammatory markers and frailty in institutionalized older men. Maturitas (2014) 79:329–33. 10.1016/j.maturitas.2014.07.014 25132319

[39] De Fanis U Wang GC Fedarko NS Walston JD Casolaro V Leng SX. T-lymphocytes expressing CC chemokine receptor-5 are increased in frail older adults. J Am Geriatr Soc (2008) 56:904–8. 10.1111/j.1532-5415.2008.01673.x 18384587 PMC2662852

[40] Semba RD Margolick JB Leng S Walston J Ricks MO Fried LP. T cell subsets and mortality in older community-dwelling women. Exp Gerontol (2005) 40:81–7. 10.1016/j.exger.2004.09.006 15664735

[41] Papanicolaou DA Wilder RL Manolagas SC Chrousos GP The pathophysiologic roles of Interleukin-6 in human disease. Annu Intern Med (1998) 13:127–37. 10.7326/0003-4819-128-2-199801150-00009 9441573

[42] Saves M Morlat P Chene G Peuchant E Pellegrin I Bonnet F Prognostic value of plasma markers of immune activation in patients with advanced HIV disease treated by combination antiretroviral therapy. Clin Immunol (2001) 99:347–52. 10.1006/clim.2001.5033 11358430

[43] Liu CK Lyass A Larson MG Massaro JM Wang N D’Agostino RB Sr Biomarkers of oxidative stress are associated with frailty: the Framingham Offspring Study. Age (Dordr) (2016) 38:1. 10.1007/s11357-015-9864-z 26695510 PMC5005887

[44] Langmann GA Perera S Ferchak MA Nace DA Resnick NM Greenspan SL. Inflammatory markers and frailty in long-term care residents. J Am Geriatr Soc (2017) 65:1777–83. 10.1111/jgs.14876 28323342 PMC5555807

[45] Ronning B Wyller TB Seljeflot I Jordhoy MS Skoylund E Nesbakken A Frailty measures, inflammatory biomarkers and post-operative complications in older surgical patients. Age Ageing (2010) 39:755–8. 10.1093/ageing/afq123 20843962

[46] Giovannini S Onder G Liperoti R Russo A Carter C Capoluongo E Interleukin-6, C-reactive protein, tumor necrosis factor-alpha as predictors of mortality in frail, community-living elderly individuals. J Am Geriatr Soc (2011) 44:735–45. 10.1111/j.1532-5415.2011.03570.x PMC4321727 21883115

[47] Tsai JS Wu CH Chen SC Huang KC Chen CY Chang CI Plasma adiponectin levels correlate positively with an increasing number of components o frailty in male elders. PLoS One (2013) 8:e56250 10.1371/journal.pone.0056250 23418545 PMC3571990

[48] Namioka N Hanyu H Hirose D Hatanaka H Sato T Shimizu S Oxidative stress and inflammation are associated with physical frailty in patients with Alzheimer’s disease. Geriatr Gerontol Int (2016) 17(6):913–8. 10.1111/ggi.12804 27296166

[49] Soysal P Stubbs B Lucato P Luchini C Solmi M Peluso R Inflammation and frailty in the elderly: a systematic review and meta-analysis. Ageing Res Rev (2016) 31:1–8. 10.1016/j.arr.2016.08.006 27592340

[50] Singh T Newman AB. Inflammatory markers in population studies of aging. Ageing Res Rev (2011) 10:319–29. 10.1016/j.arr.2010.11.002 21145432 PMC3098911

[51] Chen X Mao G Leng SX. Frailty syndrome: an overview. Clin Interv Aging (2014) 9:433–41. 10.2147/CIA.S45300 24672230 PMC3964027

